# Supplementary material for: Effect of 3d Transition Metal Atom Intercalation Concentration on the Electronic and Magnetic Properties of Graphene/MoS2 Heterostructure: A First-Principles Study
Source: Molecules. 2023 Jan 4;28(2):509. doi: 10.3390/molecules28020509 (PMC9864100; doi:10.3390/molecules28020509)
Supplement: Supplementary file 1 [file molecules-28-00509-s001.zip › molecules-2106102-supplementary.pdf]

Supporting information for

Article

Effect of 3*d* Transition Metal Atom Intercalation Concentration on the Electronic and Magnetic Properties of Graphene/MoS_2 Heterostructure: A First-Principles Study

Feng Wu [1], Zijin Wang [2], Jiaqi He [2], Zhenzhe Li [2], Lijuan Meng [1] and Xiuyun Zhang [2,*]

[1] Department of Physics, Yancheng Institute of Technology, Yancheng 224051, China
[2] College of Physics Science and Technology & Microelectronics Industry Research Institute, Yangzhou University, Yangzhou 225002, China
* Correspondence: xyzhang@yzu.edu.cn

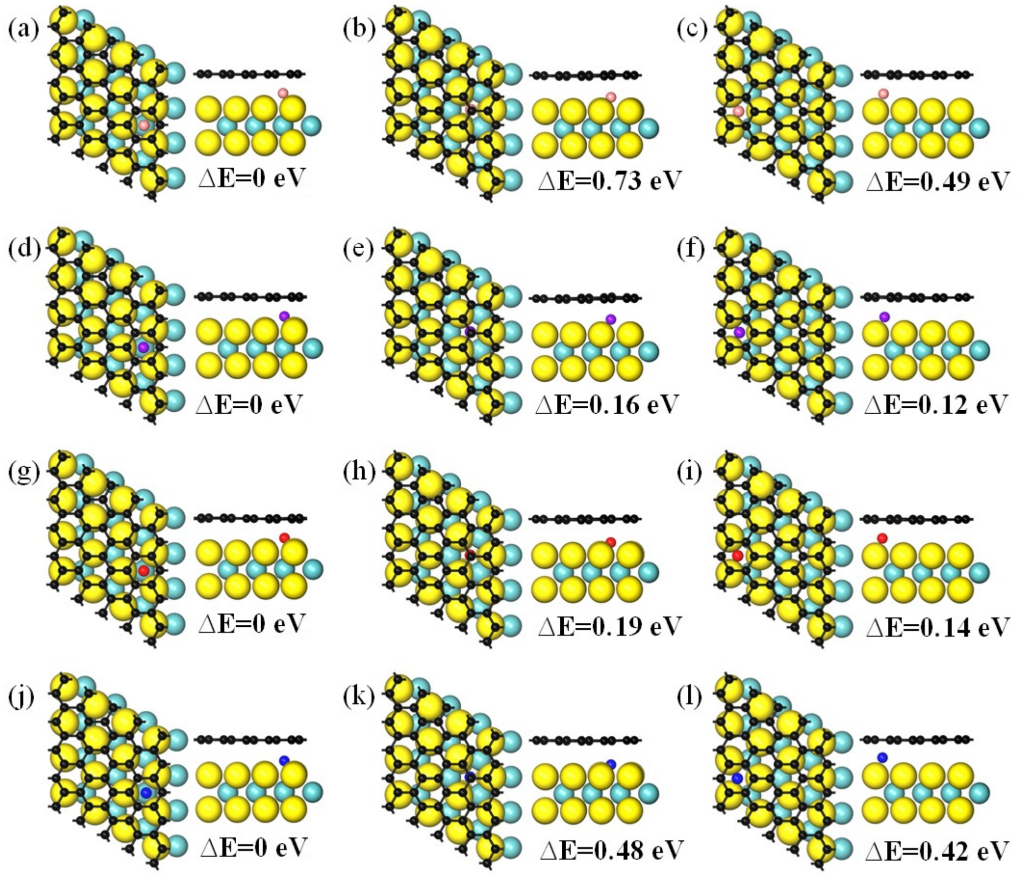

Figure S1. Optimized structures of V (a, b, c), Cr (d, e, f), Mn (g, h, i) and Fe (j, k, l) at different adsorption sites in the G/MoS_2 gap. Green, yellow, black, pink, purple, red and blue balls represent Mo, S, C, V, Cr, Mn and Fe atoms, respectively.

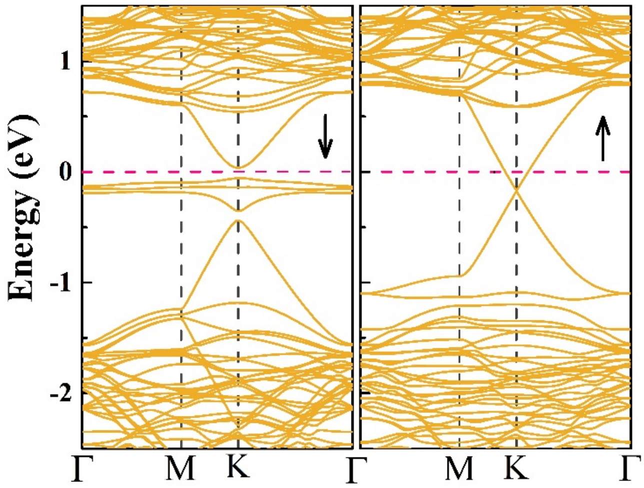

Figure S2. The spin-resolved band structure of 1Fe@(G/MoS_2) along the high-symmetry lines: minority- (left panel) and majority- (right panel) spin channels.

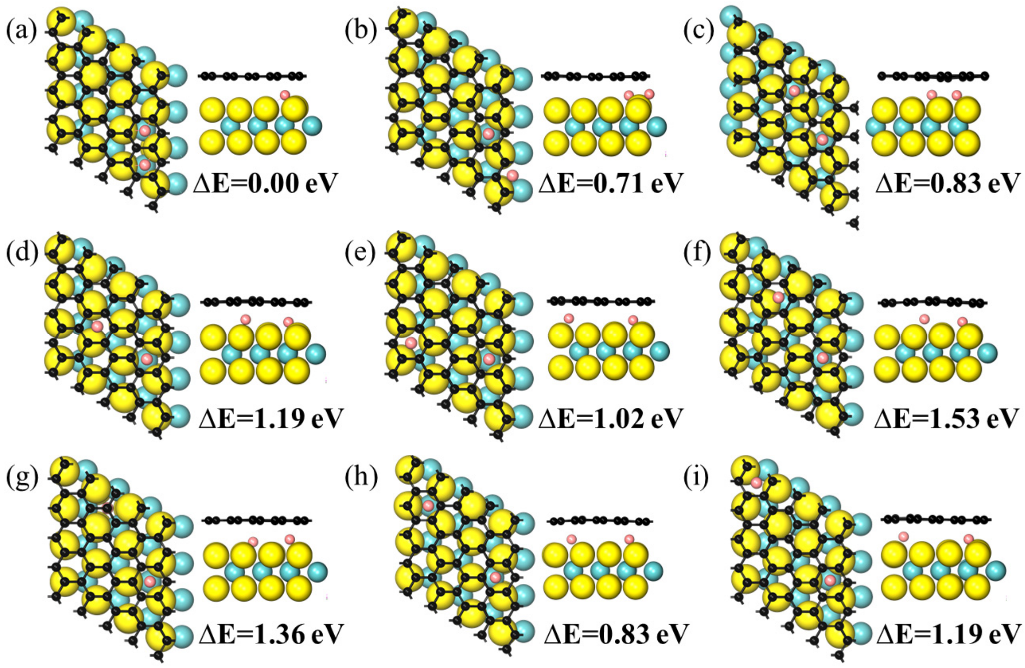

Figure S3. Optimized structures of two V atoms at different adsorption sites in the G/MoS_2 gap.

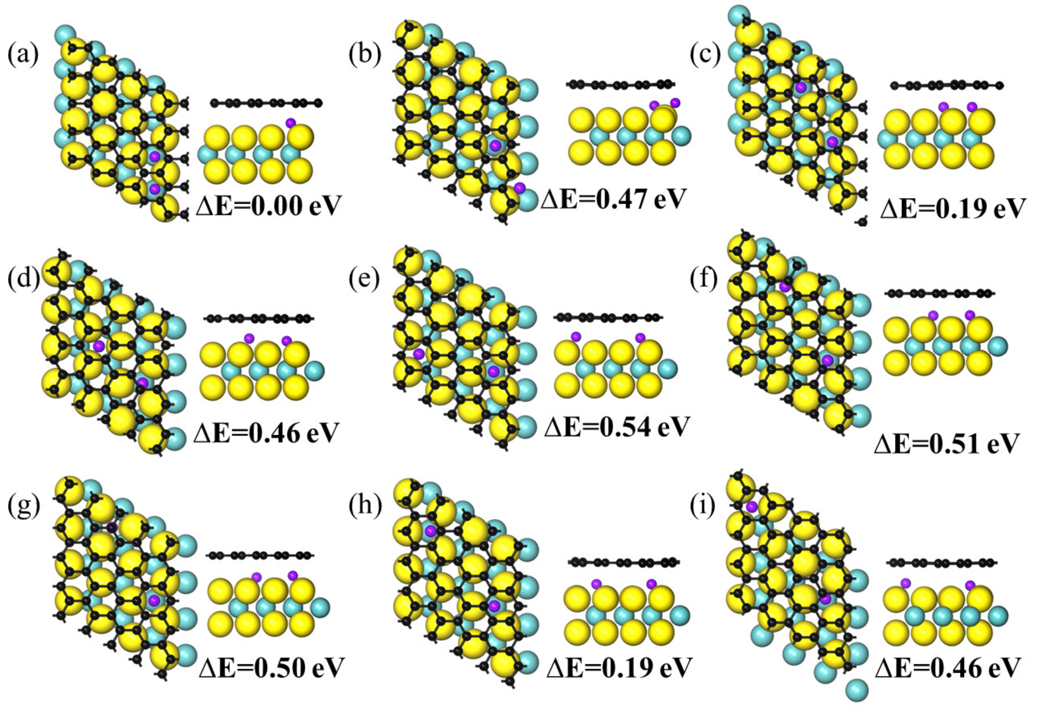

Figure S4. Optimized structures of two Cr atoms at different adsorption sites in the G/MoS_2 gap.

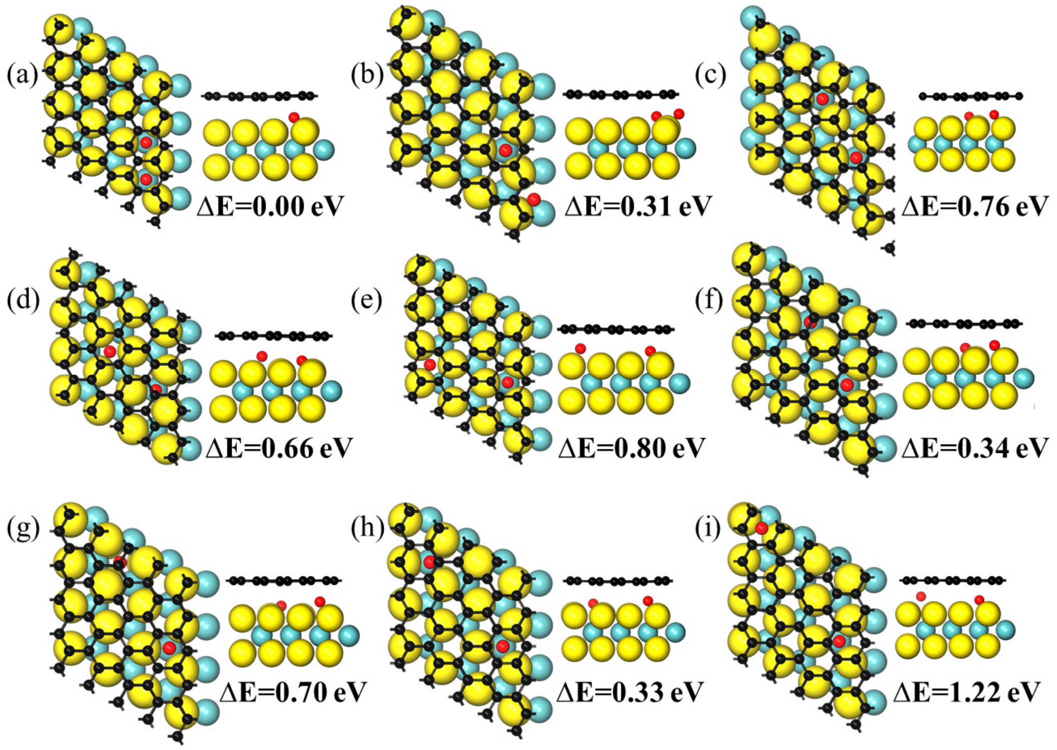

Figure S5. Optimized structures of two Mn atoms at different adsorption sites in the G/MoS_2 gap.

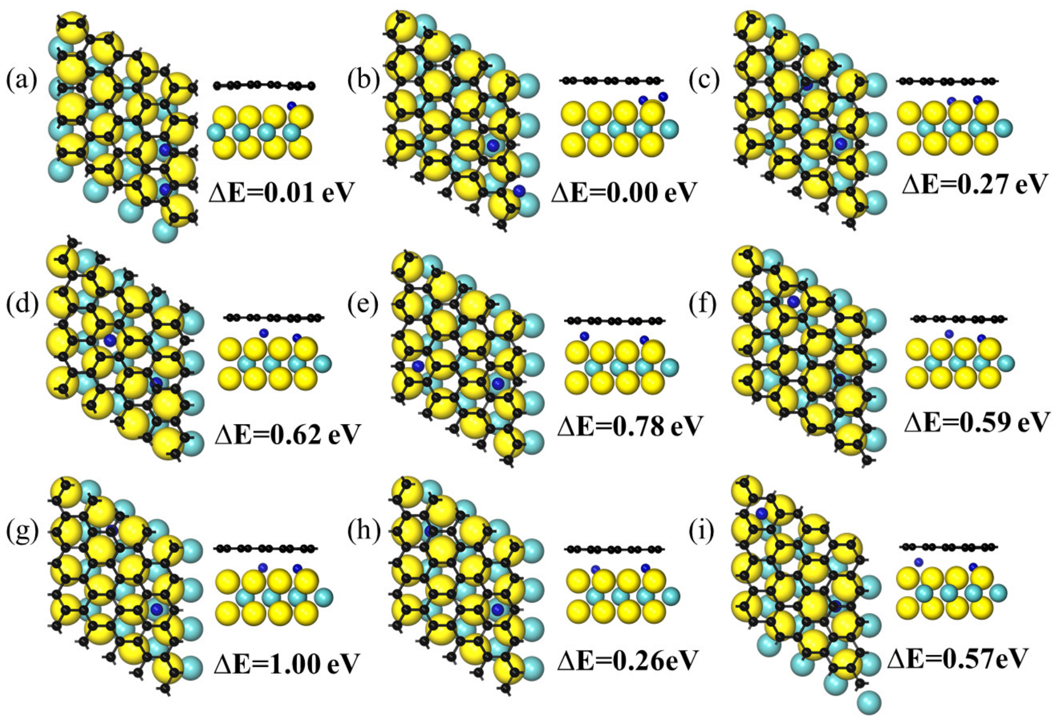

Figure S6. Optimized structures of two Fe atoms at different adsorption sites in the G/MoS_2 gap.

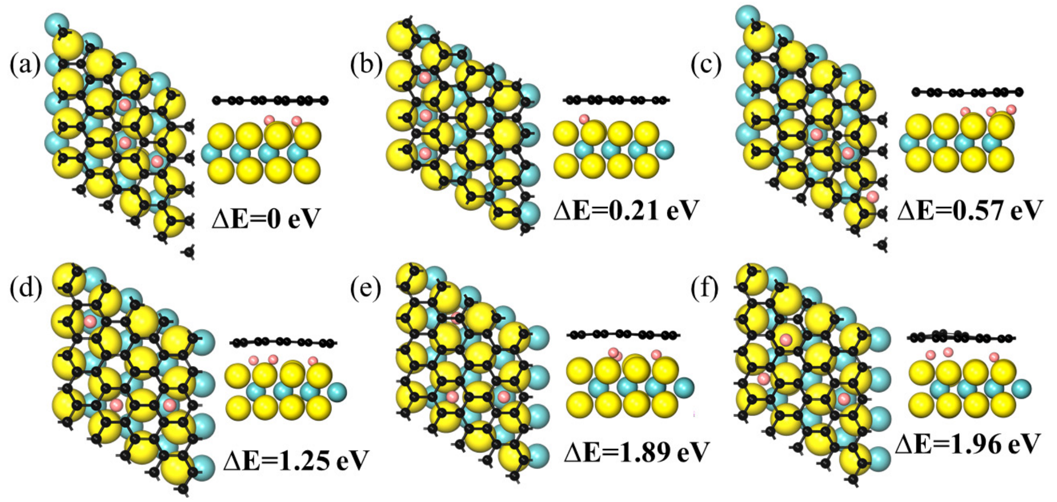

Figure S7. Optimized structures of three V atoms at different adsorption sites in the G/MoS_2 gap.

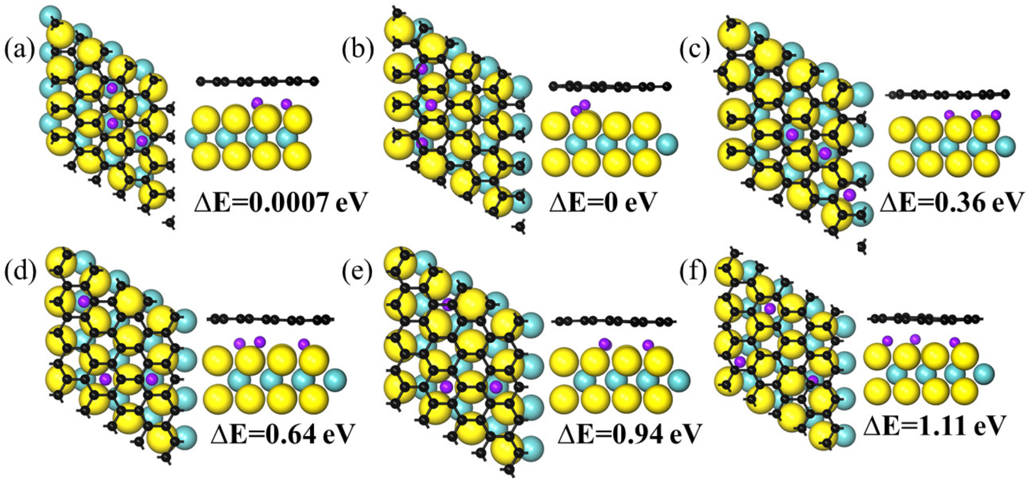

Figure S8. Optimized structures of three Cr atoms at different adsorption sites in the G/MoS_2 gap.

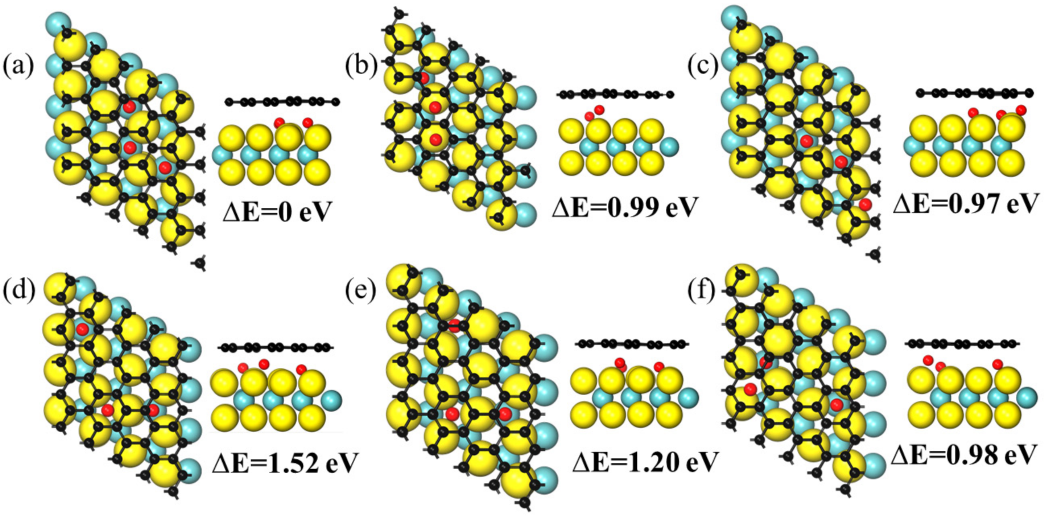

Figure S9. Optimized structures of three Mn atoms at different adsorption sites in the G/MoS_2 gap.

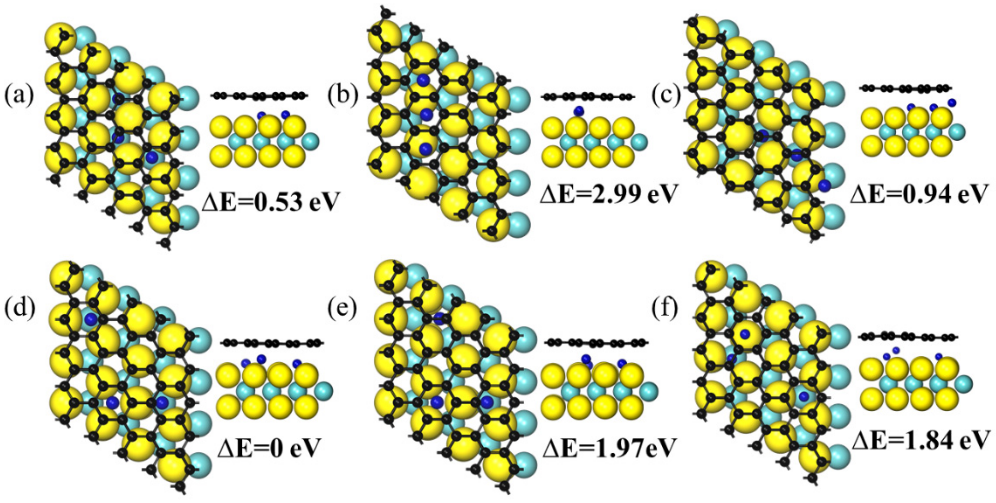

Figure S10. Optimized structures of three Fe atoms at different adsorption sites in the G/MoS_2 gap.
